# Hepatocellular adenoma classification: a comparative evaluation of immunohistochemistry and targeted mutational analysis

**DOI:** 10.1186/s13000-016-0475-5

**Published:** 2016-03-09

**Authors:** Elizabeth Margolskee, Fei Bao, Anne Koehne de Gonzalez, Roger K. Moreira, Stephen Lagana, Anthony N. Sireci, Antonia R. Sepulveda, Helen Remotti, Jay H. Lefkowitch, Marcela Salomao

**Affiliations:** Department of Pathology and Cell Biology, Columbia University Medical Center, 630 W 168th Street, VC14-238, New York, NY 10032 USA; Department of Pathology, Scripps Clinic, La Jolla, CA USA; Department of Pathology, Mayo Clinic, Rochester, MN USA

**Keywords:** Hepatocellular adenoma, Atypical hepatocellular adenoma, Liver, HCA, b-HCA

## Abstract

**Background:**

Four subtypes of hepatocellular adenomas (HCA) are recognized: hepatocyte-nuclear-factor-1α mutated (H-HCA), β-catenin-mutated type with upregulation of glutamine synthetase (b-HCA), inflammatory type (IHCA) with serum-amyloid-A overexpression, and unclassified type. Subtyping may be useful since b-HCA appear to have higher risk of malignant transformation. We sought to apply subtype analysis and assess histological atypia, correlating these with next-generation sequencing analysis.

**Methods:**

Twenty-six HCA were stained with serum amyloid A (SAA), liver fatty acid-binding protein (LFABP), glutamine synthetase (GS), and β-catenin IHC, followed by analysis with a targeted multiplex sequencing panel.

**Results:**

By IHC, 4 HCA (15.4 %) were classified as b-HCA, 11 (42.3 %) as IHCA, 9 (34.6 %) as H-HCA, and two (7.7 %) unclassifiable. Eight HCA (30.8 %) showed atypia (3 b-HCA, 4 IHCA and 1 H-HCA). Targeted sequencing confirmed *HNF1A* mutations in all H-HCA, confirming reliability of LFABP IHC in identifying these lesions. *CTNNB1* mutations were detected in 1 of 4 (25 %) of GS/β-catenin-positive cases, suggesting that positive GS stain does not always correlate with *CTNNB1* mutations.

**Conclusions:**

Immunohistochemistry does not consistently identify b-HCA. Mutational analysis improves the diagnostic accuracy of β-catenin-mutated HCA and is an important tool to assess risk of malignancy in HCA.

## Background

Hepatocellular adenomas (HCA) are rare benign liver tumors with the capacity to undergo malignant transformation. Epidemiologic studies report the prevalence of HCA is approximately 3–4 cases per 100,000 people in Europe and North America, [1] and lower in Asian countries [2]. The highest prevalence is described in females taking oral contraceptives, but other risk factors, such as anabolic steroid use, obesity and metabolic syndrome are described [2–5]. Inherited syndromes such as glycogenosis type 1, maturity-onset diabetes of the young type 3 (MODY3), and familial adenomatous polyposis have also been linked to the development of HCA [3, 5, 6]. HCA can be solitary or multiple, with “liver adenomatosis” defined as presence of multiple HCA (>10) of any size involving all liver segments [7]. The majority of HCA are benign and some regress without surgical intervention. However, serious complications can occur. Notably, hemorrhage is described in up to one fourth of patients, mainly associated with large (>5 cm) lesions. Furthermore, 9 % of HCA may transform into hepatocellular carcinoma (HCC) with risk factors including male sex, androgen use, large tumors (>5 cm) and β-catenin-mutated HCA [5]. In these complicated cases, early surgical removal may improve patient outcomes [8, 9]. Therefore, there is interest in developing diagnostics to preemptively identify high-risk cases and guide clinical management. Imaging studies may identify a proportion of HCA with relatively high sensitivities and specificities, but some cannot be accurately differentiated from HCC [10, 11]. In these cases, histological evaluation becomes essential for diagnosis.

In the last decade, four subtypes of HCA have been identified based on genotype-phenotype analyses [12]. Inactivating mutations in the *HNF1A* (*TCF1*) gene causing loss of hepatocyte nuclear factor 1α (HNF-1α) expression define H-HCA, which are characterized histologically by marked steatosis and bland hepatocyte cytology [12, 13]. A second subtype, with activating mutations of β-catenin (b-HCA), is associated with an increased risk of malignant transformation into HCC [9, 12, 14–16]. A third subtype (IHCA) is marked by inflammatory infiltrates or telangiectatic features by histology with increased serum amyloid A (SAA) and C reactive protein (CRP) expression by IHC. IHCA has been linked with activating mutations in several genes including *IL6ST, FRK, STAT3, JAK1,* and *GNAS* [17, 18]*.* Whole exome sequencing has also demonstrated that there is overlap between b-HCA and IHCA in some adenomas that harbor mutations in both the β-catenin and *IL6ST* genes [18]. Furthermore, in addition to previously reported mutations in exon 3, a smaller proportion of b-HCA carry mutations in exons 7 and 8 of *CTNNB1*; however no increased risk of malignant transformation was noted in these patients [18]. Finally, a small proportion of HCA does not appear to fall into any of the above categories and were considered unclassifiable by current testing approaches (UHCA).

Based on this classification, subtyping of HCA by immunohistochemistry has been used with some success [19]. Briefly, loss of liver fatty-acid binding protein (LFABP) staining results from *HNF-1A* mutation in H-HCA, and increased SAA immunoreactivity serves as a marker for IHCA. Upregulation of a downstream *CTNNB1* target gene, glutamine synthase (GS), is seen in b-HCA, as well as nuclear β-catenin staining. It has been postulated that the immunophenotypic subtypes closely parallel specific histologic features and molecular alterations, but limitations have been observed by numerous studies and detailed studies correlating morphology, immunohistochemical profile and mutation analysis are lacking [3, 12, 20, 21]. The aim of our study was to apply the HCA classification system based on histologic features and immunohistochemical profiles and correlate the findings with molecular analysis.

## Methods

### Case selection and histopathological evaluation

HCA cases diagnosed between January 1994 and December 2012 were retrieved from our pathology department archives. For resection specimens, representative sections of tumor and non-tumorous liver were reviewed for histological features. The presence of multiple adenomas (2 or more tumors) had been previously assessed by gross organ review and representative sections of each adenoma were examined by microscopy. For biopsies, only tumor tissue was available for review and radiology reports were reviewed to determine the presence of multiple adenomas. Retrospective chart reviews were performed to collect additional demographic data including age, gender, related medical history and clinical follow-up. The study was approved by the Institutional Review Board at Columbia University Medical Center.

Hematoxylin and eosin (H&E) stained slides, reticulin and Masson’s trichrome stains as well as immunohistochemical studies (IHC) were used to evaluate general morphologic and immunophenotypic features. All cases were reviewed by 3 pathologists (MAS, EM and FB). Tumor characteristics evaluated on routine H&E stained slides included: steatosis (mild = 0–33 %; moderate = 33–66 %; marked= > 66 % of the lesion), inflammation, sinusoidal dilatation (telangiectasia), ductular proliferation, nuclear atypia (nuclear pleomorphism, increased nuclear:cytoplasmic ratio) and architectural atypia (gland-like or acinar growth). Atypia was defined as the presence of any of the following: (1) nuclear atypia, (2) any degree of architectural atypia, and/or (3) focal loss of reticulin staining.

### Immunohistochemistry

Immunohistochemistry for LFABP (ABCAM, Cambridge, UK, 1:100 dilution), SAA (ABCAM, Cambridge, UK, 1:100 dilution), β-catenin (BD Bioscience, San Jose, CA, 1:50 dilution) and GS (Millipore, Billerica, MA, 1:2000) was performed in all cases using standard laboratory techniques in the Ventana Benchmark Ultra platform (Tucson, AZ, USA). GS IHC was scored as 0 (negative, or weak perivascular staining in <10 % of the tumor), 1+ (perivascular staining or pseudo-maplike pattern of >10 % of the tumor), and 2+ (diffuse strong staining), as previously described [19]. Pseudo-maplike GS pattern has been previously described as interconnected clusters of hepatocytes beyond perivascular lesions, connected by inconspicuous bands of positive hepatocytes [22]. β-catenin IHC was graded as 0 (membranous staining) or 1 (nuclear staining in any percentage of tumor cells). LFABP and SAA stains were scored from 0 to 2+ (Score of 0 = negative or <10 % staining, 1 + = 10–50 % staining, and 2+ = >50 % positive staining). In most cases, we used adjacent non-tumoral liver as internal negative controls, including negative SAA staining, membranous β-catenin pattern, and normal centrilobular GS positivity. CD34 immunohistochemical stains (DAKO, Carpinteria, CA, 1:200) were performed on atypical cases to evaluate for the presence of sinusoidal capillarization, as previously described [23]. In select cases, glypican-3 (Cell Marque, Rocklin, CA, 1:100) immunohistochemistry was also performed.

### Molecular analysis

Multiplex targeted DNA next generation sequencing was performed in 18 of 26 cases. DNA was extracted from frozen and/or formalin-fixed paraffin-embedded tumor tissue using QIAamp DNA Mini Kit (QIAGEN, Germany) according to manufacturer’s specifications. DNA was quantitated by fluorometry with the Invitrogen Qubit fluorometer and Quant-iT dsDNA BR Assay Kit (Life Sciences, Carlsbad, CA), as recommended by the manufacturer. The samples were sequenced using the TruSeq Amplicon Cancer Panel (MiSeq system, Illumina, CA), which covers 225 amplicons within 48 cancer-related genes, including 2 amplicons corresponding to exons 3 and 4 of *HNF1A* gene, and one amplicon representing exon 3 of *CTNNB1* gene.

Whole exome sequencing on an expanded panel of 467 tumor-specific genes, including *HNF1A*, *CTNNB1* (all exons), *IL6ST*, *STAT3*, *GNAS*, and *JAK1*, was performed in a subset of cases. This test requires higher amount of DNA and could only be performed in 11 cases with sufficient DNA (cases 2–4, 6–10, 13, 14 and 26). Target capture and enrichment were performed with the SureSelect Hybrid Capture system (Agilent Technologies, Santa Clara, CA) using custom probes. cDNA Libraries were then quantified using qPCR, diluted to 2nM and pooled for analysis on Illumina HiSeq 2500 using Illumina TruSeq v3 chemistry (Illumina, San Diego, CA).

Data deconvolution was performed using CASAVA Software (Illumina, CA). Files meeting QC metrics were used for mapping and variant calling using NextGENe Software (Softgenetics, State College, PA). Reads were aligned to the hg19 reference genome. Variant calls with allele prevalence >1 % in the 1000 Genome Project, <3 variant reads, ambiguous alignments, quality score <10, and allele frequency <10 % were excluded. Variants were cross referenced with COSMIC, PROVEAN, and SIFT prediction tools [24].

### Statistical analysis

For categorical variables, Fisher’s exact test was used. One-way Kruskal-Wallis test was used for nonparametric data, including immunohistochemical scoring. Continuous variables were compared using a two-tailed student *t*-test or one-way ANOVA, as appropriate. *P* <0.05 was regarded as statistically significant.

## Results

### Clinical characteristics

A total of 26 HCA cases (22 resections and 4 biopsies) were included in this study (male = 5, female = 21, mean age 36.2 ± 16 years). Ten patients had multiple adenomas (38.5 %) with an average number of 6 lesions per patient (range: 2–14). The average size of HCA was 8.2 ± 4.5 cm (range: 1-18 cm). Detailed clinical history and follow-up data was available in 22 cases. One or more risk factors for the development of HCA at the time of resection (e.g. use of oral contraceptives, anabolic steroid use, obesity, prior pregnancy) was identified in the majority of cases (16/23, 70 %). Among the female patients, use of oral contraceptives (OCP) was identified in 12 of 21 (57.1 %) cases. One of 5 male patients (20 %) reported anabolic steroid use. We did not identify any case of glycogen storage disease, vascular diseases or mature-onset diabetes of the young type 3 (MODY3). None of the 22 patients with follow-up data developed hepatocellular carcinoma (mean overall survival of 4.2 years). One patient died of colon cancer. The patient’s demographics and follow-up data are summarized in Table 1.

**Table 1:** Clinical information of hepatocellular adenoma cases

Case	Sex	Age (years)	Multiple adenomas	Avg. Size (cm)	Survival (days)	Risk Factors
1	F	66	Y	6.4	365	N
2	M	22	N	13	365	N
3	M	24	N	18	182.5	N
4	F	7	N	15.1	4962	N
5	F	11	Y	11	3235	N
6	M	34	N	14.9	612	Obesity^a^
7	F	40	N	8	547.5	OCP
8	F	32	Y	2.5	n/a	OCP
9	F	38	N	4.4	n/a	OCP
10	F	39	N	7.1	4745	OCP
11	F	19	N	2.5	1460	n/a
12	F	29	N	6.3	1104	OCP
13	F	17	Y	7.6	2988	N
14	F	54	N	10	246	Prior pregnancy
15	F	45	Y	9	939	OCP
16	M	25	N	8.5	730	Androgen use
17	F	39	Y	10	1095	OCP, prior pregnancy
18	F	34	Y	8	n/a	OCP, prior pregnancy
19	F	42	N	5.5	n/a	OCP
20	F	58	Y	4	2190	OCP, obesity, prior pregnancy
21	F	41	Y	4.5	2190	OCP
22	F	75	N	5.5	deceased	n/a
23	M	32	N	18	1713	n/a
24	F	38	Y	1	7190	N
25	F	52	N	7.2	1255	N
26	F	29	N	6.3	1095	OCP

*Abbreviations*: *F* female, *M* male, *N* absent, *Y* Present, *n/a* data not available, *OCP* oral contraceptive use, ^a^Obesity is defined as BMI > 30

### Histopathological and immunohistochemical analysis

**Fig. 1:**
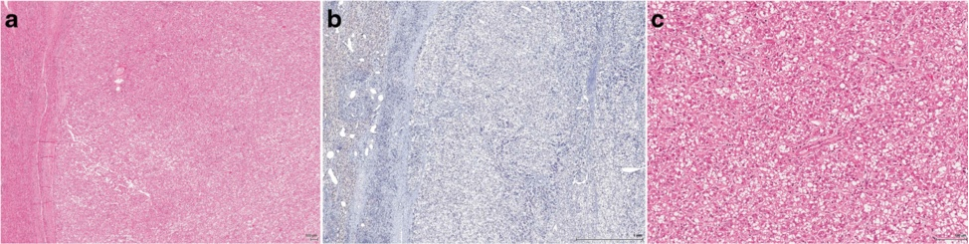
Histological features of HNF-1a inactivated hepatocellular adenoma (H-HCA). **a**, H-HCA with steatosis and **b**, loss of LFABP expression (H&E and IHC X 20). **c**, higher magnification showing marked steatosis (H&E x100)

**Fig. 2:**
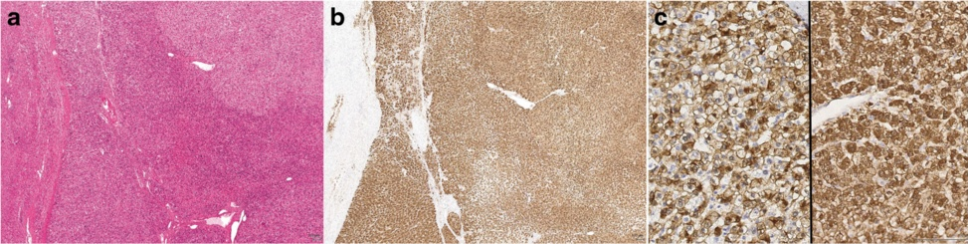
Histological features of beta-catenin activated hepatocellular adenoma (b-HCA). **a**, Lower power micrograph showing border of b-HCA (*middle and right*) with normal liver (*left*) (H&E x20). **b**, Diffuse GS expression in b-HCA (*right*) in contrast to the focal perivenular staining in the normal liver tissue (*left*) (IHC X 20). **c**, diffuse and strong GS staining (*right panel*) vs. focal β-catenin nuclear staining (*left panel*) in b-HCA (IHC X 200)

**Fig. 3:**
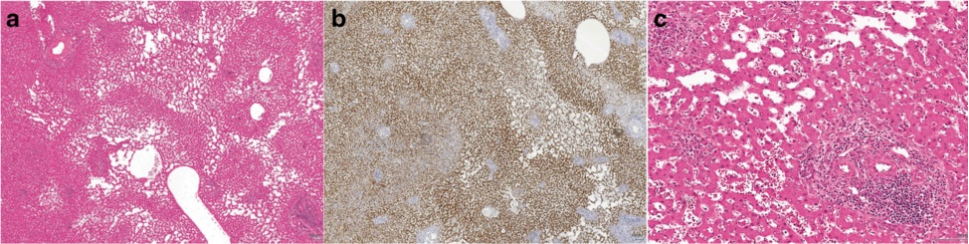
Histological features of inflammatory hepatocellular adenoma (IHCA). **a**, IHCA with telangiectasia and focal inflammation (H&E X 20). **b**, Diffuse serum amyloid-A expression within the tumor (IHC X 20). **c**, Higher magnification micrograph highlighting inflammation (*lower right*) and telangiectasia (*upper left*) (H&E X 100)

First, we attempted to classify each adenoma based on IHC pattern, as previously described [19]. Briefly, LFABP-negative cases were classified as H-HCA (Fig. 1). HCA with strong and diffuse GS staining (score 2+) and/or β-catenin nuclear staining, regardless of the SAA staining status, were categorized as b-HCA (Fig. 2). The remaining HCA with SAA positivity (scores 1+ to 2+) were classified as IHCA (Fig. 3).

By applying the above criteria, we identified 4 b-HCA (15.4 %), 11 IHCA (42.3 %), and 9 H-HCA (34.6 %). Two cases (7.7 %) were negative or weakly positive for GS, negative for SAA, and positive for LFABP, and were therefore unclassifiable (Table 2).

**Table 2:** Histological, immunohistochemical and mutational analysis of hepatocellular adenomas

	Morphological features		Immunohistochemistry				Mutational Analysis	Initial Classification^b^	Final Classification^c^
Case	Morphology	Atypical features	SAA	LFABP	B-cat	GS			
1	0	NuA, Lret	1	2	0	2	CTNNB1 c.114_119del; p.38_40del^1^ TP53 c.T751A; Ile251Phe^1,2^	b-HCA	b-HCA
2	T	NuA, AG, Lret	1	2	1	2	IL6ST c.C657A; Pro216His^1,2,3^	b-HCA	*IHCA*
3	T	0	0	2	1	2	IL6ST c.554_568del; p.185_190del^1,3^	b-HCA	*IHCA*
4	0	NuA, AG, Lret	0	2	1	2	nm**	b-HCA	*favor b-HCA*
5^a^	0	AG, Lret	1	2	0	1	n/a	IHCA	n/a
6	T, I	NuA, AG, Lret	1	2	0	1	nm**	IHCA	IHCA
7	T, I, DR	NuA	2	2	0	1	CTNNB1 c.T133C; Ser45Pro^1,3^ IL6ST c.566_577del; p.189_193del^1,3^	IHCA	*bIHCA*
8	T, I	0	2	2	0	0	nm**	IHCA	IHCA
9	T, I, DR	0	2	2	0	0	nm**	IHCA	IHCA
10	T, I, DR	0	2	2	0	1	nm**	IHCA	IHCA
11	T, DR	NuA, Lret	2	2	n/a	0	nm	IHCA	IHCA
12	T	0	2	2	0	0	n/a	IHCA	n/a
13^a^	T, I, S (20 %)	0	2	2	0	1	nm**	IHCA	IHCA
14	T, I, DR, S (60 %)	0	2	2	n/a	0	nm**	IHCA	IHCA
15^a^	T, I, DR	0	2	2	0	0	n/a	IHCA	n/a
16	S (30 %)	NuA	2	0	0	2	HNF1A c.620G > A; Gly207Asp^1,3^	H-HCA	H-HCA
17	S (20 %)	0	2	0	0	1	HNF1A c.857A > G;Tyr286Cys^1^	H-HCA	H-HCA
18	S (40 %)	0	0	0	0	0	HNF1A c.842 T > A;Leu281Gln^1^	H-HCA	H-HCA
19	S (70 %)	0	0	0	0	0	n/a	H-HCA	n/a
20	S (80 %)	0	0	0	0	0	HNF1A c.710A > G; Asn237Ser^1,3^	H-HCA	H-HCA
21	S (70 %)	0	2	0	0	0	HNF1A c.710A > T; Asn237Lys^1,3^	H-HCA	H-HCA
22	S (10 %)	0	2	0	n/a	0	n/a	H-HCA	n/a
23	I, S (30 %)	0	2	0	0	0	n/a	H-HCA	n/a
24	T, I, S (50 %)	0	0	0	0	0	n/a	H-HCA	n/a
25^a^	S (10 %)	0	0	2	0	1	n/a	UHCA	n/a
26	I	0	0	2	0	0	nm**	UHCA	UHCA

*Abbreviations*: *B-HCA* β-catenin activated hepatic adenoma, *IHCA* inflammatory HCA, *bIHCA* HCA with mixed IHCA and b-HCA features, *H-HCA* HNF1A-inactivated HCA, *UHCA* unclassifiable HCA, *SAA* serum amyloid A, *LFABP* liver fatty acid-binding protein, *b-cat* β-catenin, *GS* glutamine synthetase, *0* absent, *T* telangiectasia, *I* inflammation, *DR* ductular reaction, *S* Steatosis (% fat), *NuA* nuclear atypia, *AG* acinar growth, *Lret* focal loss of reticulin stain, *n/a* not available, *nm* no mutation/deletion/insertion identified in HNF1A and CTNNB1 exon 3, nm** no mutation/deletion/insertion identified in HNF1A, IL6ST, STAT3, GNAS, JAK1 and CTNNB (expanded gene analysis)

^a^Only biopsy specimen available; ^b^Classification based on IHC alone; ^c^Classification following molecular evaluation (italic font is used to mark cases that were re-classified after molecular analysis); 1Deleterious alteration by PROVEAN prediction; 2 variant previously described in HCC; 3 variant previously described in HCA

By immunohistochemistry, we found significant overlap between the b-HCA and IHCA subtypes. Focal SAA staining (1+, <50 % of cells) was present in 2 out of 4 b-HCA (cases 1 and 2). Conversely, 5 IHCA with 1+ or 2+ SAA staining showed weak, heterogeneous GS expression (score 1+) (cases 5–7, 10 and 13). We chose not to classify these as mixed β-catenin/inflammatory HCA based solely on immunohistochemistry because none of them demonstrated strong positivity for both GS and SAA. Cases with loss of LFABP did not show any staining for GS, except for one H-HCA case with strong (2+) GS staining (Table 2, case 16). In addition, strong (2+) SAA staining was present in 5 of 9 H-HCA (LFABP-negative) cases and might have resulted from proximity to areas of tumor necrosis or hemorrhage within the tumor, findings identified in at least 3 cases. Nuclear β-catenin staining was present in 3 of 5 (60 %) GS positive (2+) cases, but was limited to rare to few cells corresponding to less than 5 % of total tumor area and predominantly located in perivascular areas (Fig. 2c).

During the interpretation phase of this study, we encountered two technical difficulties. First, we found that LFABP IHC can sometimes be faint in the non-neoplastic liver resulting in an inconspicuous gradient between native liver and tumor. In such cases, LFABP staining was repeated in multiple tumor sections in order to obtain acceptable results. Second, the few cases with nuclear β-catenin positivity were difficult to evaluate since nuclear positivity could only be appreciated in less than 5 % of the tumor cells.

### Molecular analysis

Results of targeted DNA sequencing analysis were correlated with the classification obtained by immunohistochemical analysis (Table 2). *HNF1A* mutations were identified in all analyzed H-HCA (5/5, 100 %) and in no other HCA subtype (*p* value = 0.001). The codon alterations were Gly207Asp, Asn237Ser, Asn237Lys, Leu281Gln and Tyr286Cys, 3 of them previously reported in H-HCA [18].

**Fig. 4:**
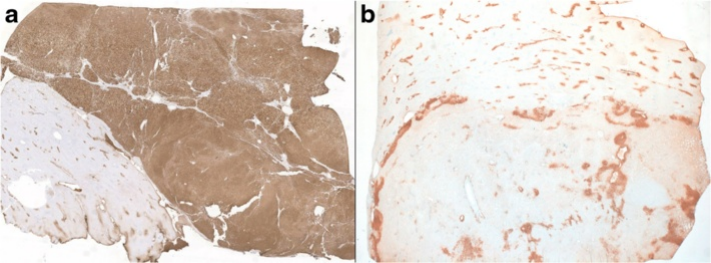
Patterns of glutamine synthetase staining in HCA. **a**, Diffuse GS staining in *CTNNB1* wild-type tumor (case 4) (upper right, x 10). **b**, GS staining of bIHCA carrying both *CTNNB1* and *IL6ST* alterations (case 7) shows perivascular and focal pseudomap-like pattern (lower aspect, x 10). As internal positive control, note the normal centrilobular pattern of GS staining of the adjacent non-neoplastic liver

*CTNNB1* gene alterations were identified in 2 cases: one b-HCA harboring both a *CTNNB1* gene deletion (c.114_119del; p.38_40del) and a *TP53* gene mutation (Ile251Phe) (case 1); and a second case originally classified as IHCA based on IHC results (ie. strong diffuse SAA positivity and focal GS staining), found to have a missense *CTNNB1* mutation (Ser45Pro). This case also carried an *IL6ST* mutation (c.566_577del; p.189_193del) and was thus reclassified as a mixed β-catenin/inflammatory HCA (bIHCA) (case 7, Fig. 4b). *CTNNB1* alterations in exons 7 and 8 were not identified in the 11 tumors analyzed by whole-exome sequencing. Six tumors classified as IHCA due to 2+ SAA staining had no mutations in the IHCA-related pathways.

A total of 4 cases were reclassified following molecular analysis. One case was the bIHCA described above. The other 3 tumors, initially classified as b-HCA (2+ GS staining), were reclassified because molecular analysis demonstrated no abnormalities in the *CTNNB1* gene. Two of them carried *IL6ST* gene alterations (*IL6ST* c. C657A; Pro216His and *IL6ST* c.554_568del; p.185_190del) and were reclassified as IHCA. The third case had no alterations in *CTNNB1, HNF1A, IL6ST, STA3, GNAS or JAK1* genes and final classification could not be confirmed by molecular studies (Fig. 4a).

Following classification by molecular testing (Table 2, last column), we analyzed the histological features of each subtype (Figs. 1, 2 and 3). All cases classified as H-HCA by IHC showed steatosis of variable degree. IHCA (*n* = 9) showed features of telangiectasia (*n* = 9, 100 %), focal inflammation (*n* = 6, 66.7 %) and ductular reaction (*n* = 4, 44.4 %). Mild to moderate steatosis was occasionally seen IHCA (*n* = 2, 22.2 %). Telangiectasia, a feature commonly associated with IHCA, was seen in the bI-HCA (case 7). No cases classified as b-HCA, IHCA or UHCA showed marked steatosis.

In rare occasions, limited DNA concentration or DNA degradation may limit detection of mutations and deletions in tumor samples. To circumvent this issue, DNA was obtained from both frozen and formalin-fixed tumor samples and sequenced using two different platforms. This approach was feasible in 12 of our 18 analyzed samples and yielded reproducible sequencing results.

By our sequencing methods, LFABP IHC captured all H-HCA cases and was 100 % specific, whereas combination of GS and β-catenin IHC predicted β-catenin mutations with a sensitivity of 50 %, specificity of 75 %, and PPV of 20 %. Correlation between histological features, IHC results and molecular analysis showed that LFABP loss and tumor steatosis were significantly associated with H-HCA (*p* = 0.001 and 0.036, respectively). Not surprisingly, telangiectasia was preferentially present in IHCA (*p* = 0.0002) (Table 3).

**Table 3:** Correlation between histological features and molecular classification of HCA

	H-HCA (*n* = 5)	b-HCA/bIHCA (*n* = 2)	IHCA (*n* = 9)	UHCA (*n* = 1)	*p* value
Telangiectasia	0	50 % (1)	100 % (9)	0	0.0002
Inflammation	20 % (1)	50 % (1)	67 % (6)	100 % (1)	NS
Ductular Reaction	0	50 % (1)	44 % (4)	0	NS
Steatosis (mean %)	48 %	0	9 %	0	0.04
Atypical features	20 % (1)	100 % (2)	44 % (4)	0	NS
SAA positive (1+/2+)	60 % (3)	100 % (2)	89 % (8)	0	NS
GS positive (1+/2+)	40 % (2)	100 % (2)	56 % (5)	0	NS
LFABP loss	100 % (5)	0	0	0	0.0001

*Abbreviations*: *SAA* serum amyloid A, *LFABP* liver fatty acid-binding protein, *GS* glutamine synthetase, *b-HCA* beta-catenin activated hepatic adenoma, *bIHCA* HCA with mixed IHCA and b-HCA features, *IHCA* inflammatory HCA, *H-HCA* HNF1A inactivated HCA, *UHCA* unclassifiable HCA, *NS* not statistically significant (*p* value >0.05)

### Evaluation of atypia in HCA

**Fig. 5:**
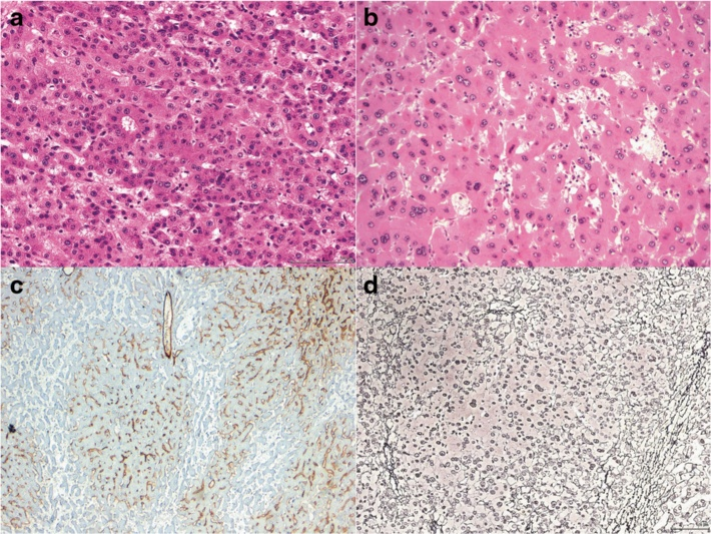
Histological atypia in HCAs. **a**, pseudoacinar formation (H&E X 200). **b**, nuclear atypia (H&E X 200). **c**, capillarization of the HCA on CD34 immunohistochemical stain (X 100). **d**, focal loss of reticulin by reticulin silver stain (X 100)

Eight cases (30.8 %) displayed one of more atypical features (Table 2, example in Fig. 5). By molecular testing, these cases were classified as b/b-IHCA (2 of 2, 100 %), IHCA (4 of 9, 44.4 %), H-HCA (1 of 5, 20 %), and UHCA (1 of 2, 50 %). Nuclear atypia was present in 7 cases (87.5 %). Gland-like or acinar growth was seen in 4 of 8 cases (50 %). Reticulin staining showed focal (<1 mm) loss of reticulin framework in 6 cases (75 %). By CD34 immunostaining, focal or patchy sinusoidal capillarization was seen in 6 cases (75 %), but diffuse CD34 staining classically described in malignant hepatocellular lesions was not identified. Glypican-3 immunohistochemistry was negative in all atypical cases. Atypical HCA were more frequently present in male patients, but this result did not reach statistical significance (3 of 5 males [60 %] vs. 5 of 21 females [23.8 %], *p* = 0.28). Similarly, patient age was not significantly different in patients with atypical HCA (mean age 28 vs. 39.9 years, *p* = 0.07).

## Discussion

Hepatocellular adenomas have a small risk of malignant transformation with estimated rates of less than 5 % [3, 25–27]. Recent advances in the morphologic and molecular classification of HCA identified b-HCA as the subtype most commonly associated to malignant transformation. This finding warrants the use of robust and reliable approaches to identify such tumors [12, 18]. In particular, the status of *CTNNB1* mutations in HCA may offer crucial information regarding management of some of these patients.

The current HCA classification scheme is based on the tumor’s morphologic and immunohistochemical characteristics and was originally validated with *CTNNB1* and *HNF1A* gene sequencing [28]. More recently, high-throughput sequencing of a large HCA cohort led to the discovery of novel genetic alterations, [17, 18] including new hot-spots in *CTNNB1* gene in the b-HCA subtype, and defects in the IL-6/JAK/STAT3 pathway in IHCA. Our study is one of the first to use a comprehensive targeted next-generation sequencing panel to evaluate the mutational status of HCA and correlate with immunohistochemistry results.

As expected, our study demonstrated *HNF1A* gene mutations in all analyzed H-HCA cases, confirming high accuracy of this marker in the diagnosis of H-HCA. However, our results suggest limited reliability of GS and β-catenin immunohistochemistry in predicting β-catenin mutations. The utility of both markers has been challenged by other researchers [10, 21, 22, 29, 30]. Lagana *et al.* showed strong GS positivity in over 50 % of HCA and Joseph *et al.* described patchy to diffuse GS staining in 23 % of IHCA, but no correlation with molecular studies were available in these studies [22]. In the present study, seven of 12 GS-positive cases displayed faint perivascular staining or heterogeneous positivity. Further molecular evaluation performed in 5 tumors failed to demonstrate *CTNNB1* alterations in 4 cases, one of which was a biopsy specimen (case 13). These somewhat controversial staining patterns and their correlation with β-catenin activation have not been fully characterized and may be a result of localized metabolic alterations within the tumor [20–22, 31].

In addition, we identified strong GS positivity in four β-catenin wild-type tumors, including one H-HCA. We speculate whether these tumors may have activation of an alternative pathway resulting in GS expression. Berry *et al.* described strong GS staining in a peliotic HCA and suggested that vascular flow alterations and hepatic parenchymal remodeling may explain increased GS expression [31, 32].

In our study, GS staining failed to identify 1 of 2 confirmed *CTNNB1*-mutated HCA. Low GS expression in b-HCA has been previously reported in HCA carrying the Ser45Pro mutation [33]. Interestingly, the same β-catenin mutation has been described in HCC and was not associated with nuclear β-catenin staining, suggesting that some *CTNNB1* alterations may not disrupt GS and β-catenin expression patterns [34]. None of the 11 cases evaluated by whole-exome sequencing carried large *CTNNB1* deletions or mutations in exons 7 or 8, the latter described in a minority of bHCA and previously shown to activate β-catenin in HCC cell lines [18]. Caution is warranted in such cases as they show weak and patchy GS staining and lack nuclear staining of β-catenin [18].

Following molecular analysis, four tumors were reclassified. One case phenotypically classified as IHCA was reclassified as bIHCA because it carried both a β-catenin mutation and an *IL6ST* alteration. Three cases initially categorized as b-HCA lacked *CTNNB1* gene alterations. Two of them were re-subtyped as IHCA following identification of *IL6ST* gene alterations, all of which previously described in IHCA [17, 35]. The remaining case carried no mutations in *HNF1A* or any of the IHCA-related genes. In this challenging case, the diagnosis of a β-catenin-activated HCA is favored, but the lack of molecular evidence for *CTNNB1* alterations suggests alternative mechanisms of β-catenin activation and GS overexpression in the tumor [36]. This adenoma was originally resected from a 7-year-old patient with no known medical conditions, who remains disease-free after over 13 years of her surgery. HCA occurrence in preadolescence is extremely rare and little is known about the natural history and pathogenesis of such lesions. Six adenomas immunophenotypically compatible with IHCA lacked identifiable mutations in IHCA-associated genes, a finding previously described by others [18, 27].

A large percentage of HCA showed atypical features (30.8 %) but no correlation with a specific HCA type was identified. Other histological features, such as mild steatosis and telangiectasia, were not reliable predictors of HCA subtype as defined by IHC and molecular analysis. In addition, we identified significant immunohistochemical overlap between the b-HCA and IHCA subtypes. Some of these “overlap” lesions, with immunohistochemical features of both IHCA and b-HCA, have been previously described as carrying *CTNNB1* activating mutations [18]. Both β-catenin-mutated cases showed SAA staining and atypical histological features, but alterations in the inflammatory pathway were only present in one case.

There is a strong association between b-HCA and hepatocellular carcinoma at the time of diagnosis or during follow-up [19]. In our study, none of our cases progressed to HCC. One possible explanation is the fact that our group consisted predominantly of females on oral contraceptives, features generally associated with lower risk of malignant transformation [18]. Interestingly, one b-HCA with atypia harbored a *CTNNB1* exon 3 deletion (Gly38_Thr40del) and a *TP53* mutation (Ile251Phe), the latter located in a known ‘hotspot’ for cancer-related alterations and previously described in HCC [37]. For the purposes of this study, we favor a very well-differentiated HCC arising from b-HCA. Evason *et al.* described HCC-related genetic alterations in β-catenin-activated adenomas suggesting that some of these lesions more likely represent low-grade hepatocellular carcinomas [38]. No other HCC-related driver mutations were identified in our study set.

Immunohistochemistry remains the mainstay for HCA classification due to its relative reliability and rapid turnaround time, especially when differentiating HCA from other lesions, such as focal nodular hyperplasia and hepatocellular carcinoma [22, 39]. However, given the inherent variability in GS staining and the low sensitivity of β-catenin IHC, we believe that molecular testing should be used in all HCA cases with equivocal staining patterns, especially in high-risk patients. Our findings are limited by the small sample size and must to be confirmed by a systematic review of a larger number of well-characterized HCA including correlation of each immunohistochemical pattern with mutational profiles detected by molecular analysis.

## Conclusion

In summary, while LFABP immunohistochemistry is reliable in identifying H-HCA, GS and β-catenin stains are weak predictors of β-catenin mutations. Our molecular analysis demonstrates that the currently proposed IHC criteria may be somewhat oversimplified. Until other reliable and specific immunohistochemical markers of malignant transformation in HCA are identified, molecular testing of LFAPB-positive HCA may be the best strategy to identify some of the lesions requiring further clinical management. In this context, next-generation sequencing offers a straightforward, highly sensitive, and accurate option for biopsies with equivocal immunohistochemical results.
